# Inhibitory Effects of Bacterial Silk-like Biopolymer on Herpes Simplex Virus Type 1, Adenovirus Type 7 and Hepatitis C Virus Infection

**DOI:** 10.3390/jfb13010017

**Published:** 2022-02-02

**Authors:** Esmail M. El-Fakharany, Marwa M. Abu-Serie, Noha H. Habashy, Nehal M. El-Deeb, Gadallah M. Abu-Elreesh, Sahar Zaki, Desouky Abd-EL-Haleem

**Affiliations:** 1 Proteins Research Department, Genetic Engineering and Biotechnology Research Institute (GEBRI), City of Scientific Research and Technological Applications, Alexandria 21934, Egypt; 2 Medical Biotechnology Department, Genetic Engineering and Biotechnology Research Institute (GEBRI), City of Scientific Research and Technological Applications, Alexandria 21934, Egypt; marwaelhedaia@gmail.com; 3 Biochemistry Department, Faculty of Science, Alexandria University, Alexandria 21511, Egypt; noha.habashi@alexu.edu.eg; 4 Biopharmacetical Products Research Department, Genetic Engineering and Biotechnology Research Institute, City of Scientific Research and Technological Applications, Alexandria 21934, Egypt; nehalmohammed83@gmail.com; 5 Environmental Biotechnology Department, Genetic Engineering and Biotechnology Research Institute (GEBRI), City of Scientific Research and Technological Applications, Alexandria 21934, Egypt; g_abouelrish@yahoo.com (G.M.A.-E.); saharzaki@yahoo.com (S.Z.); abdelhaleemm@yahoo.de (D.A.-E.-H.)

**Keywords:** bacterial silk, biopolymer proteins, antiviral, HSV-1, adenovirus, HCV

## Abstract

Bacterial polymeric silk is produced by *Bacillus* sp. strain NE and is composed of two proteins, called fibroin and sericin, with several biomedical and biotechnological applications. In the current study and for the first time, the whole bacterial silk proteins were found capable of exerting antiviral effects against herpes simplex virus type-1 (HSV-1), adenovirus type 7 (AD7), and hepatitis C virus (HCV). The direct interaction between bacterial silk-like proteins and both HSV-1 and AD7 showed potent inhibitory activity against viral entry with IC_50_ values determined to be 4.1 and 46.4 μg/mL of protein, respectively. The adsorption inhibitory activity of the bacterial silk proteins showed a blocking activity against HSV-1 and AD7 with IC_50_ values determined to be 12.5 and 222.4 ± 1.0 μg/mL, respectively. However, the bacterial silk proteins exhibited an inhibitory effect on HSV-1 and AD7 replication inside infected cells with IC_50_ values of 9.8 and 109.3 μg/mL, respectively. All these results were confirmed by the ability of the bacterial silk proteins to inhibit viral polymerases of HSV-1 and AD7 with IC_50_ values of 164.1 and 11.8 μg/mL, respectively. Similarly, the inhibitory effect on HCV replication in peripheral blood monocytes (PBMCs) was determined to be 66.2% at concentrations of 100 μg/mL of the bacterial silk proteins. This antiviral activity against HCV was confirmed by the ability of the bacterial silk proteins to reduce the ROS generation inside the infected cells to be 50.6% instead of 87.9% inside untreated cells. The unique characteristics of the bacterial silk proteins such as production in large quantities via large-scale biofermenters, low costs of production, and sustainability of bacterial source offer insight into its use as a promising agent in fighting viral infection and combating viral outbreaks.

## 1. Introduction

Natural products have gathered special attention because of the surviving biodiversity of flora worldwide and the luxury of obtaining extracts and crude forms from these sources with the help of technological innovation [1,2]. In addition to collagen, polymeric silk is one of the most plentiful naturally derived macromolecular protein polymers obtained mainly from animal origins [3]. More than 120,000 metric tons of silks are manufactured worldwide annually, and the main manufacturers are located in China, India, and Japan [4]. Polymeric silk is a natural protein of importance in medical applications and commercial life. Increased uses of bacterial polymeric silk-like proteins are expected in the near future for several reasons such as low cost–effect, rapid secretion, and modest product recapture. Silk proteins consist mostly of two types of proteins in a core–shell type shape: fibroin composes the core and is enclosed by the glue-like sericin, which cements itself to the fibroin fibers. Sericin, a major constituent of silk proteins, is selectively removed as waste material from silk fiber throughout the silk manufacturing process to make silk more lustrous. Commonly, the removal of sericin from fiber leads to the formation of industrial byproducts that are disposed of as waste materials, causing environmental pollution and constituting a huge waste of natural resources. Thus, sericin will need to be recovered and recycled in an adequate manner to provide social and significant economic benefits [5]. Sericin is easily hydrolyzed although it is considered an insoluble protein in cold water. Sericin is an useful protein owing to its unique features such as oxidation resistance, antibacterial potential, UV resistance, capacity to absorb and release moisture easily, and tyrosine kinase inhibitory activity [6,7,8,9,10,11,12]. Sericin is also a glycoprotein that possesses many biological characteristics, including antioxidation, immunomodulation, and inhibition of elastase and tyrosinase activities. Recently, sericin was found to reduce reactive oxygen species (ROS), protect normal cells from oxidative damage caused by UVB radiation, and inhibit tumor progression, being a strong antibacterial candidate [13,14]. Meanwhile, researchers suggest that viruses are the most abundant biological entities on the planet [15]. Currently, millions of people are infected with different viruses, and many of these people are not receiving treatment or vaccination such as infection with retroviruses [16,17]. Enveloped viruses consist of a protein coat called a capsid that surrounds central genetic material, which is either DNA or RNA and is unable to replicate without a host cell. Therefore, to survive, viruses must infect cells and use these cells to replicate themselves. In the manner of doing this, they can kill these cells and cause damage to the host organism, which is why viral infections can make people ill. Hepatitis C virus (HCV), adenovirus type 7 (ADV7), and herpes simplex virus type 1 (HSV1) infections are universal serious diseases, and they lead to several hepatic sequels, death, and genital herpes [18,19,20]. 

The high cost of viral disease treatment and/or vaccinations leads to patients using alternative remedies as a traditional medicine for viral infection control. There are many examples of traditional medicine such as the use of camel milk for HCV treatment [21], as camel milk contains many important proteins that play a crucial role in viral prevention [22,23,24]. In addition, there are many microbial metabolites are used as effective compounds in viral treatment, including mushroom and cyanobacterial lectins [25,26,27,28]. Medical usage of natural bioactive ingredients as a main source of treatment to date is well known, even considering the great contribution of chemotherapeutic drugs to modern therapy. However, there is no information on the potential effects of the silk proteins of animal or bacterial origin on viral diseases such as infection with HCV, HSV-1, and AD7. If either one of these proteins could be shown to be effective against pathogenic viruses, it might be a more affordable source for use in medicinal/pharmacological applications, thus contributing to providing a sustainable production and reduction in medical costs.

In this study, we evaluated the antiviral inhibitory activity of whole bacterial silk proteins in an obtained silk of nonanimal origin, which was produced by *Bacillus* sp. strain NE (a petroleum-originated bacteria) [10,29]. This antiviral effect was measured to investigate the ability of the whole bacterial silk proteins as a nonanimal silk source, produced by *Bacillus* sp. strain NE, to inhibit the infection of HSV-1, ADV7, and HCV, viruses that cause a severe challenge to the worldwide public health system owing to their limited vaccination or symptomatic treatment.

## 2. Materials and Methods

### 2.1. Whole Bacterial Polymeric Silk-like Protein Isolation and Silk-like Protein Preparation

The bacterial polymeric silk-like proteins were produced by *Bacillus* sp. strain NE as previously identified according to Kamoun et al. [29]. In brief, *Bacillus* sp. strain NE was inoculated in 100 mL nutrient broth (SNB) medium (containing 500 mL of nutrient broth and 500 mL mineral salt solution per liter). A 500 mL salt solution was prepared by mixing various salts (K_2_HPO_4_, 5.0 g; KH_2_PO_4_, 20 g; NaCl, 0.1 g; (NH_4_)_2_SO_4_, 30 g; FeSO_4_·7H_2_O, 0.01 g; CaCl_2_·2H_2_O, 0.01 g; MgSO_4_·7H_2_O, 0.2 g; MnSO_4_·7H_2_O, 0.2 g; MnSO4·H_2_O, 0.002 g) CaCl_2_·2H_2_O, 0.01 g; glucose, 0.03% (*w*/*v*) (Fluka Chemie, Gillingham, UK); and yeast extract, 0.03% (*w*/*v*) [30], and it was incubated at 30 °C overnight in a shaker incubator at 150 rpm. Then, 5 mL of bacterial culture was inoculated in 100 mL TSM (containing per liter: 5 g of KNO3, 30 g trypticase soy broth, and 20 g of L-glutamic acid) production medium and incubated for 5 days under the same conditions. The formed exo-biopolymer was precipitated by centrifugation of the cultured media for 30 min at 8000 rpm. After the crude supernatant was concentrated by a rotary evaporator and dialyzed against distilled water at 4 °C overnight, about 30 mL cold ethanol was added to 10 mL of dialyzed concentrate. Then, the precipitate was mixed with 10% cetylpyridinium chloride (CPC) during gentle stirring. The obtained broth was left under room conditions for several hours to obtain the polymeric precipitate by centrifugation at 5000 rpm for 30 min. The precipitate was dissolved in 0.5 M of sodium chloride to obtain the polymeric broth. The polymeric broth was lyophilized after washing three times with cold ethanol to obtain the purified bacterial polymeric-like silk proteins [29].

### 2.2. Serum Sample Collection

HCV-4a patient serum samples used in this investigation were obtained from the Institute of Medical Research, Alexandria, Egypt. Serum samples were stored at −80 °C prior to viral inoculation experiments. The patients’ written consent and approval for this study were obtained from the institutional ethics committee.

### 2.3. Human Peripheral Blood Mononuclear Cell Separation

Peripheral blood mononuclear cells (PBMCs) were separated from whole human blood using gradient centrifugation by Ficoll-Paque Plus (MP Biomedicals, Illkirch, France) as reported by Lohr et al. [31]. Briefly, whole blood sample was 5 times diluted using freshly prepared PBS and then overlaid dropwise on Ficoll. The monolayer of PBMCs was separated using gradient centrifugation at 2000 rpm for 30 min. The recovered cells were collected and washed 3 times using PBS.

### 2.4. Cytotoxicity Assays

African green monkey kidney epithelial cell line (Vero cells) was obtained from American Type Culture Collection (ATCC) via VACSERA (Cairo, Egypt). Vero cells were cultured in DMEM medium and used as adenovirus type 7 (ADV7) and herpes simplex virus type 1 (HSV-1) host cells. While PBMCs (hepatitis C virus (HCV) host cells) were cultured in RPMI medium. Silk protein cytotoxicity was tested on both Vero cells and PBMCs using MTT assay [32,33]. Briefly, both Vero cells and PBMCs were seeded in 96-well cell culture plates at densities of 10^4^ and 10^5^ cells/well and incubated at 37 °C in 5% CO_2_ for 24 h. After incubation, serial dilutions of the tested silk protein were incubated with Vero and PBMCs for 72 h. Serial dilutions of the HSV-1 and ADV7 standard drugs (ribavirin and acyclovir, respectively) were incubated with Vero cells for 72 h. At the end of incubation, 20 μL of 5 mg/mL MTT (Sigma, St. Louis, MO, USA) was added to each well and the plates were incubated at 37 °C for 3 h. After discarding MTT solution, 100 µL DMSO (dimethyl sulfoxide) was added and the dye intensity was quantified using the automated ELISA microplate reader adjusted to 570 nm to quantify the cell viability [26,34]. The effective safe concentration (EC_100_) doses (at 100% cell viability) of the tested protein were estimated by the GraphPad Prism 9 InStat software.

### 2.5. The Antiviral Activity of Silk Proteins against ADV7 and HSV-1

Ten-fold dilutions of ADV7 and HSV-1, separately, were incubated with a monolayer of Vero cells in 96-well plates for 2 h in a 5% CO_2_ incubator. At the end of incubation, the unabsorbed viruses were aspirated and replaced with DMEM containing 10% FBS. Then, the plates were incubated in a 5% CO_2_ incubator for 3 days. MTT assay was used to determine cell viability (%), as described above, in the infected and uninfected cells to calculate TCID_50_ (50% tissue culture infectious dose) using the formula of the Ramakrishnan method [35].

#### 2.5.1. MTT and RT-qPCR Assays for Investigation of Antiviral Activity of Silk Protein

The utilized 100TCID_50_ (100 times the TCID_50_) viral inocula (10^−4^ and 10^−3^ for ADV7 and HSV-1, respectively) were used to test the mode of antiviral action of silk protein. For assessment of the direct virucidal effect, different concentrations of silk protein and standard drugs were incubated for 2 h with HDV7 and HSV-1, before being added to the Vero cell monolayer in a 96-well plate. The adsorption inhibition effect was evaluated by pretreating the host cells with serial dilutions of the tested protein and standard drugs for 2 h, and then the viruses were added to Vero cells for another 2 h. For the antireplicative effect, serial doses of silk protein and standard drugs were added after incubating Vero with viruses for 2 h. After 3 days, MTT assay was used as described above to determine the percentage of cell lysis inhibition for each protein concentration to calculate the IC_50_ (using GraphPad InStat software) at which silk protein causes 50% viral inhibition. 

Moreover, quantitative real-time PCR (qPCR) was used to confirm the results of silk protein antiviral mode action obtained from the MTT assay. Following the seeding of Vero cells in 6-well culture plates for 24 h, the above-mentioned experiments were repeated using 100 μg/mL and 10 μg/mL of silk protein against ADV7 and HSV-1, respectively, and compared to the standard drugs with the same concentrations. After 72 h incubation in CO2, the untreated and treated infected Vero cells, in each well, were collected for total DNA extraction using Qiagen extraction kit. TaqMan-based real-time PCRs (CFX, BIO RAD) were performed in accordance with [36,37]. The ADV7 qPCR primers were 5’-GAGGAGCCAGATATTGATATGGAATT-3’ and 5’-AATTGACATTTTCCGTGTAAAGCA-3’ with the probe 5’-6-carboxyfluorescein (FAM)-AAGCTGCTGACGCTTTTTCGCCTGA-6-carboxytetramethylrhodamine (TAMRA)-3’. For HSV-1 qPCR, primers were 5’-CATCACCGACCCGGAGAGGGAC-3’ and 5’-GGGCCAGGCGCTTGTTGGTGTA-3’ and the probe was 5’-FAM-CCGCCGAACTGAGCAGACACCCGCGC-6-TAMRA-3’. The reaction mixture contained Taq polymerase (0.05 U/μL) and reaction buffer (0.4 mM of dNTP, 250 nM probe, 400 nM forward/reverse primers, and 4 mM MgCl_2_). PCR program started with 95 °C for 15 min, followed by 45 cycles of 95 °C for 10 s, 55 °C for 30 s, and 72 °C for 20 s. Viral load was estimated using standard curve.

#### 2.5.2. Evaluation of the Inhibitory Impact of Silk Protein on DNA Polymerase Activity of ADV7 and HSV-1

The inhibitory effect of silk protein on viral DNA polymerase activity was determined using acid-precipitated radioactivity. The reaction mixture of ADV polymerase consisted of 25 mM Tris-HCl pH 7.8, 7 mM MgCl_2_, 1 μg aphidicolin, 10 mM DTT, activated DNA, and 40 μM deoxynucleotides with 1µ Ci radiolabeled [α-^32^P]dATP [38]. Meanwhile, the reaction mixture of HSV polymerase contained 50 mM Tris-HCl pH 8, 0.5 μg/mL albumin, 100 mM ammonium sulfate, 8 mM MgCl_2_, 0.5 mM DTT, and 100 μM deoxynucleotides with 1µ Ci radiolabeled [^3^H-dTTP] [39,40]. The above-mentioned mixtures of ADV7 and HSV-1 were incubated with serial concentrations of silk protein for 30 and 60 min, respectively. After reaction termination by acid precipitation, radioactivity was measured using a scintillation counter.

#### 2.5.3. The Antiviral Activity of Silk Protein against HCV

The inhibitory effect of silk protein on HCV in the isolated PBMCs of healthy donors was recorded. A nontoxic concentration of silk protein was simultaneously incubated with the virus (sterile filtered infected serum) for 90 min. Then, cells were infected with 100 μL of the cotreated virus for 72 h at 37 °C and 5% CO2. At the end of incubation, the infected cells were washed three times with 1 mL of PBS, and the total RNA was extracted using Qiagen Kit. The positive control of the infected untreated cells and the negative control of uninfected cells were included in the experiment.

#### 2.5.4. Quantification of HCV Genomic and Antigenomic RNA Strands Using RT-PCR

To quantify HCV genomic and antigenomic RNA strands (minus strand), reverse transcription nested PCR was carried out as described previously and with minor changes [31]. Briefly, the reaction was carried out in 25 μL with 20 U of reverse transcriptase (Clontech, Mountain View, CA, USA) with 400 ng of the total extracted PBMC RNA, 40 U of RNAsin (Clontech, Mountain View, CA, USA), dNTP (Promega, Madison, WI, USA, at final concentration of 0.2 mmol/L) and the reverse primer 1CH (for plus strand, 50 pmol) or forward primer 2CH (for minus strand, 50 pmol). The reaction was developed for 60 min at 42 °C and then denatured for 10 min at 98 °C. The amplification of highly conserved 5′-UTR sequences was completed using two PCR rounds (with two pairs of nested primers). The first-round amplification was conducted in 50 μL reaction mixture having 2CH forward primer and P2 reverse primer (50 pmol from each), as well as dNTPs (0.2 mmol/L). RT reaction mixture (10 μL) was used as a template with 2 U of Taq DNA polymerase (Promega, Madison, WI, USA). The thermal cycling protocol was as follows: 94 °C for 1 min, 55 °C for 1 min, and 1 min at 72 °C for 30 cycles. The second amplification cycle was similar to the first one, except for use of the nested reverse primer D2 and forward primer F2 (50 pmol). Primer sequences for the 5′ HCV noncoding (NC) region amplification were as follows: 1CH: 5′-ggtgcacggtctacgagacctc-3′, 2CH: 5′-aactactgtcttcacgcagaa-3′, P2: 5′-tgctcatggtgcacggtcta-3′, D2: 5′-actcggctagcagtctcgcg-3′, and F2: 5′-gtgcagcctccaggaccc-3′. To overcome the false detection of negative-strand HCV RNA and known variations in PCR competence, specific control assays and rigorous standardization of the reaction were completed: (1) cDNA was synthesized without RNA templates to avoid product contamination, (2) cDNA synthesis was synthesized without RTase to avoid Taq polymerase RTase activity, and (3) cDNA synthesis and PCR step were completed without any reverse or forward primers to avoid the contamination from mixed primers. In addition, cDNA synthesis was conducted using one primer followed by heat inactivation of RTase activity at 95 °C for 1 h, in an effort to reduce the false presence of negative strands before the addition of the second primer. Finally, the RT-PCR was conducted using the final PCR product based on the SYBR Green I dye and LightCycler fluorimeter [41]. An external standard curve was done using 10-fold serial dilutions of a modified synthetic HCV 5′ NC RNA [42].

#### 2.5.5. Quantification of the Induced ROS Using Oxidized DCFDA and Flow Cytometry

ROS and oxidative damage are assumed to have a vital role in many human diseases. Using a cell-permeable fluorescent and chemiluminescent probe, 2’-7’-dichlorodihydrofluorescein diacetate (DCFH-DA), we quantified the induced ROS in HCV-infected and treated cells using flow cytometry [43]. Briefly, after cellular treatment with the nontoxic dose of silk protein, all silk-protein-treated, HCV-infected, and control PBMCs were incubated with DCFH-DA at a final concentration of 10 μM for 30 min at 37 °C and 5% CO2. After incubation, cells were washed with prewarmed PBS and suspended in FAC buffer solution. The intensity of fluorescence was quantified by flow cytometry (Partec GmbH, Germany), and the sample redox state was monitored by checking the increase in fluorescence that could be measured at 530 nm when the sample is excited at 485 nm.

### 2.6. Protein Modeling and Validation

Based on our previous data [10,29], there is a great similarity between the *Bombyx mori* silk protein and the *Bacillus* silk protein. The analysis of the amino acid constituents showed that the block protein is rich in Ala-Pro-Gly, while the molecular weight (Mwt) of fibroin protein H-chain was around 400 kDa, which exceeded that of the natural silk of *Bombyx mori* [44]. The bacterial silk-like nanofibers gave an adequate and uniform ribbon-shaped structure with an average nanofiber diameter of around 110 nm. Meanwhile, the block silk-like protein exhibited high thermal degradation monitored at 140–373 °C, which is close to that of natural silkworms like *Bombyx mori* [45]. In our recently published work [30], the fibroin H-chain exhibited a band at ~400 kDa, while the fibroin L-chain exhibited a band at ~35 kDa and P25 proteins exhibited a band at ~30 kDa. However, sericin proteins exhibited bands at 95, 66, 16, and 6.5 kDa. The *Bombyx mori* silk fibroin P25 protein and sericin 1A’ were used in the current study for the docking analysis due to the availability of their full sequences in the NCBI database. In addition, the Mwt of these proteins is similar to those of our bacterial silk proteins. Therefore, the current study used *Bombyx mori* silk fibroin p25 and sericin 1A’ for the in silico analysis due to the availability of their full sequences in the NCBI database. The Protein Data Bank (PDB, Long Island, NY, USA, https://www.rcsb.org/ (accessed on 6 October 2021) was used to derive the 3D structures of HCV-NS5B (PDB: 3FQK, 576 res, chain A and B) and HSV1 DNA polymerase (PDB: 2GV9, 1193 res, chain A and B). However, the PDB of the unavailable 3D structures of silk fibroin, silk sericin, and human ADV7 DNA polymerase were generated by the Swiss-Model protein-modeling server (https://swissmodel.expasy.org/ (accessed on 13 October 2021) [46]. The amino acid sequences of silk fibroin (Accession: AAL83649, 262 amino acids), sericin 1A’ (Accession: BAD00699, 722 amino acids), and ADV7DNA polymerase (Accession: ASK85767, 1193 amino acids) were retrieved from the NCBI protein database and then submitted to the Swiss-Model for analysis. The validation of the generated structural models was done by the PROCHEK Ramachandran plot [47,48]. The theoretical molecular weight values of the two *Bombyx mori* silk protein subunits were predicted from the protein amino acids by the EXPASY server (https://web.expasy.org/compute_pi/ (accessed on 14 October 2021) [49] to compare them with those obtained from the *Bacillus* strain.

### 2.7. Molecular Docking Analysis

The predicted mechanism of the silk sericin and silk fibroin inhibitory impact on the activity of HCV-NS5B and the DNA polymerase of HSV1 and ADV7 was assessed using molecular docking. The docking of the highest C-score proposed 3D structure models of silk sericin and silk fibroin, individually with each of the studied viral polymerases, was established by the GRAMM-X Protein-Protein Docking Server (http://vakser.compbio.ku.edu/resources/gramm/grammx/ (accessed on 14 October 2021) [50]. Then, the binding pocket atoms of the created docked structural complexes were visualized and analyzed using the Discovery Studio 2020 Client program (v20.1.0.19295, Dassault Systèmes, Vélizy-Villacoublay, France).

### 2.8. Assessment of the Binding Affinity in the Docked Complexes

The PDBePISA (Proteins, Interfaces, Structures, and Assemblies) website (https://www.ebi.ac.uk/msd-srv/prot_int/pistart.html (accessed on 15 October 2021) [51] was used to analyze the interface of the docked complexes. The numbers of interface residues and hydrogen bonds, along with the change in Gibbs free energy (ΔG, solvation free energy) gained upon interface formation, are provided by this platform. The ΔG value indicates the docked complex’s binding affinity.

### 2.9. Active Site Prediction

The PDBsum web-based database (http://www.ebi.ac.uk/pdbsum (accessed on 19 October 2021) [48] was used in the current study to retrieve the active site residues of the HCV and HSV1 polymerases. The PDB IDs of these viral enzymes were required by this website; hence, it provides structural data on PDB database entries. However, the active site residues of ADV7 polymerase, which has no PDB ID, were predicted using the GASS-WEB server (https://gass.unifei.edu.br/ (accessed on 23 October 2021) [52].

### 2.10. Statistical Analysis

All data are expressed as mean ± standard error of the mean (SEM). The unpaired two-tailed Student’s *t*-test of SPSS 16.0 was used. Statistical differences were expressed as *p*-value < 0.05 *, <0.001 **, <0.0001 ***.

## 3. Results

### 3.1. Cytotoxicity Assay

**Figure 1:**
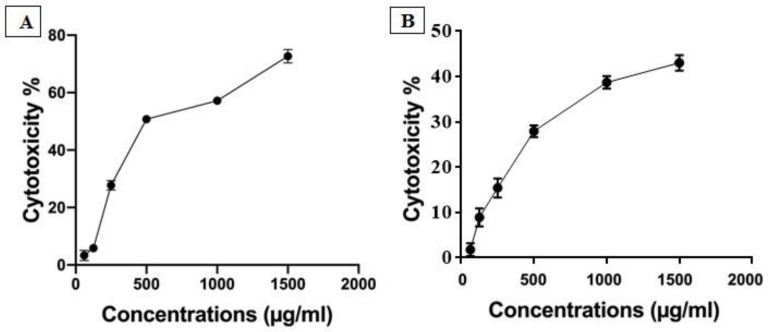
The safety patterns of silk protein on PBMCs (**A**) and Vero cells (**B**); different concentrations of silk protein (from 1500 to 62.5 µg/mL) were tested on PBMCs to detect the nontoxic dose and IC_50_ value of silk protein.

Cytotoxicity assay was used to quantify both IC_50_ and nontoxic dose of silk protein on PBMCs and Vero cells using MTT assay protocol. The obtained results indicated that the nontoxic dose (EC_100_) of silk protein on PBMCs and Vero cells reached 100 µg/mL with IC_50_ determined to be 460 and 743.3 µg/mL, respectively. So, we selected a 100 µg/mL dose for completion of the antiviral assay (Figure 1). The recorded safe doses of silk protein, ribavirin, and acyclovir on Vero cells were 1000, 284.3, and 266.1 μg/mL, respectively.

### 3.2. Antiviral Assays

#### 3.2.1. Antiviral Assays on ADV7 and HSV1

**Figure 2:**
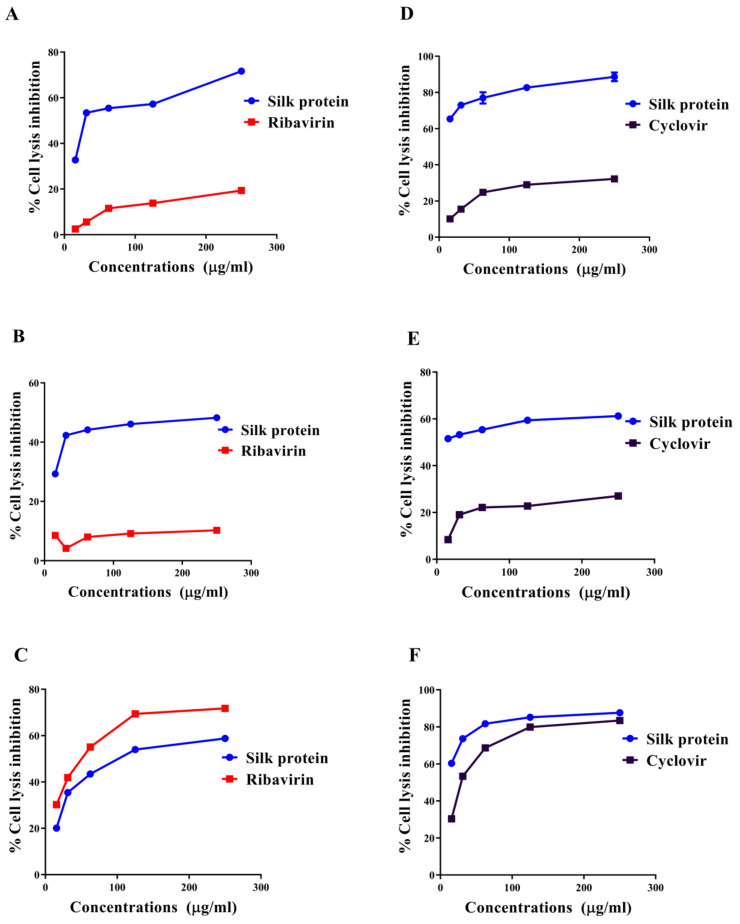
Percentage lysis inhibition of adenovirus (ADV7)- and herpes simplex virus (HSV-1)-infected Vero cells after treatment with silk protein with modes of action illustrated. (**A**) Direct virucidal, (**B**) antiadsorption, and (**C**) antireplication effects of silk protein against ADV7 in comparison with standard drug (ribavirin) in the term of % cell lysis inhibition. (**D**) Direct virucidal, (**E**) antiadsorption, and (**F**) antireplication activity of silk protein against HSV1 in comparison with standard drug (acyclovir) in the term of % cell lysis inhibition. All data are expressed as mean ± SEM.

The antiviral mode of silk protein was investigated, at 100TCID_50_ (10^−4^ and 10^−3^ of ADV7 and HSV-1, respectively), by quantifying the percentages of cell lysis inhibition and viral elimination using MTT and qPCR, respectively. This was achieved by preincubating the tested proteins with viruses before application to host cells (direct virucidal), pretreatment of host cells before adding viruses (antiadsorption), and addition after infection of host cells (antireplicative). Figure 2 illustrates that silk protein inhibited cell lysis in a dose-dependent manner. From these curves (Figure 2), IC_50_ values, representing the concentration at which 50% inhibition of cell lysis occurs, were calculated for each antiviral mode, and the lowest value refers to the most effective action mode(s) of this protein for combating ADV7 and HSV-1. The estimated IC_50_ values of silk protein for ADV7 inactivation were 46.4 ± 0.5 μg/mL, 222.4 ± 1.0 μg/mL, and 109.2 ± 2.1 μg/mL by direct virucidal, antiadsorption, and antireplicative modes of action, respectively. The values of IC_50_ for HSV-1 inactivation were 4.1 ± 0.7 μg/mL, 12.5 ± 0.1 μg/mL, and 9.8 ± 0.3 μg/mL by direct virucidal, antiadsorption, and antireplicative modes of action, respectively. These IC_50_ values and the highest percentage of cell lysis inhibition indicate that silk protein can inhibit virus-mediated cell lysis mainly via a direct virucidal effect with the lowest antiadsorption effect on both viruses. This protein exhibited a comparable antireplicative effect on both viruses. Standard drugs (ribavirin and acyclovir) only exhibited antireplicative effects with IC_50_ equivalent to 48.9 ± 1.6 μg/mL and 30.9 ± 0.7 μg/mL, respectively, but IC_50_ values for other effects could not be determined because their maximum safe concentration did not reach 50%. Accordingly, the anti-ADV and anti-HSV efficacy of silk protein is mainly achieved by direct virucidal and antireplicative manner, respectively. Therefore, these modes were selected for the following evaluation of its antiviral activity using more specific parameters. 

**Figure 3:**
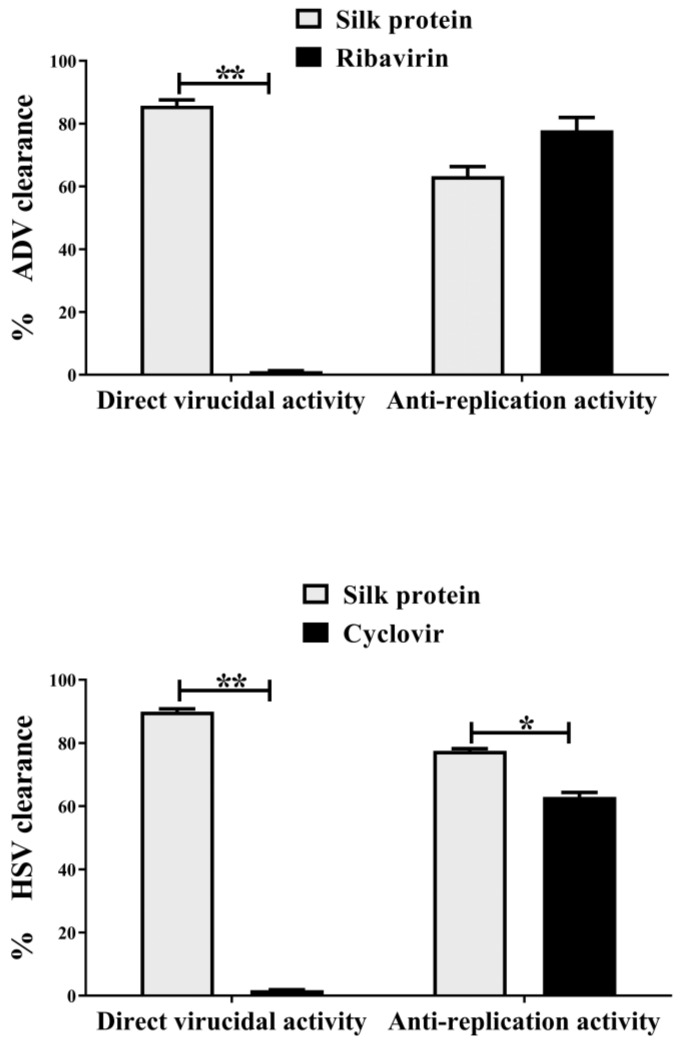
Percentage of adenovirus (ADV7) and herpes simplex virus (HSV-1) eliminated by direct virucidal and antireplication potentials of silk protein in comparison with standard drugs (ribavirin and acyclovir). All data are expressed as mean ± SEM and considered significantly different at *p* < 0.05 *, *p* < 0.005 **.

Data of qPCR for cellular virus contents supported MTT results of cell lysis inhibition. As shown in Figure 3, this protein eliminated 85.7% and 89.9% of ADV7 and HSV1, respectively, by direct virucidal activity, while its antireplication effect caused viral elimination by 63.2% and 77.5%, respectively. On the other hand, standard drugs achieved viral elimination (77.9% and 62.9%, respectively) only via their antireplicative effect. Figure 3 also shows that silk protein had significantly stronger direct ADV7 and HSV1 virucidal effect and anti-HSV1 replicative potential than standard drugs. Meanwhile, no significant difference in anti-ADV7 replicative activity was recorded between silk protein and ribavirin.

**Figure 4:**
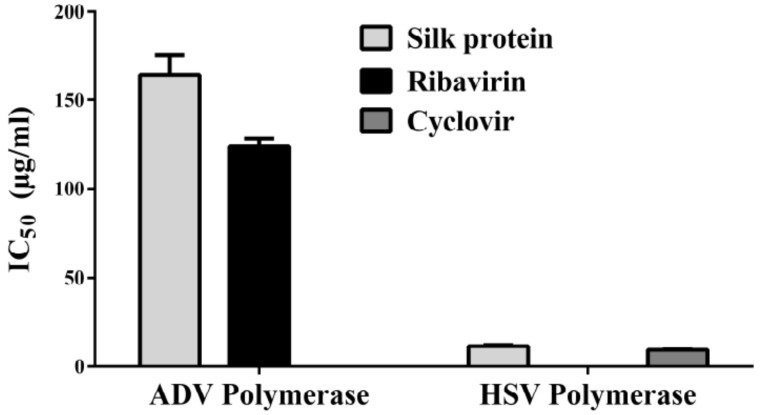
IC_50_ values of silk protein for inhibiting DNA polymerases of ADV and HSV. All data are expressed as mean ± SEM.

The antireplicative activity of silk protein was further assessed by estimating the IC_50_ for inhibition of viral DNA polymerases (Figure 4). It was found that silk protein can inhibit these viral polymerases at 164.1 ± 11.1 μg/mL and 11.8 ± 0.6 μg/mL for ADV7 and HSV1, respectively, with no significant difference when compared to standard drugs (Figure 4).

#### 3.2.2. Anti-HCV Activity of Silk Protein

**Figure 5:**
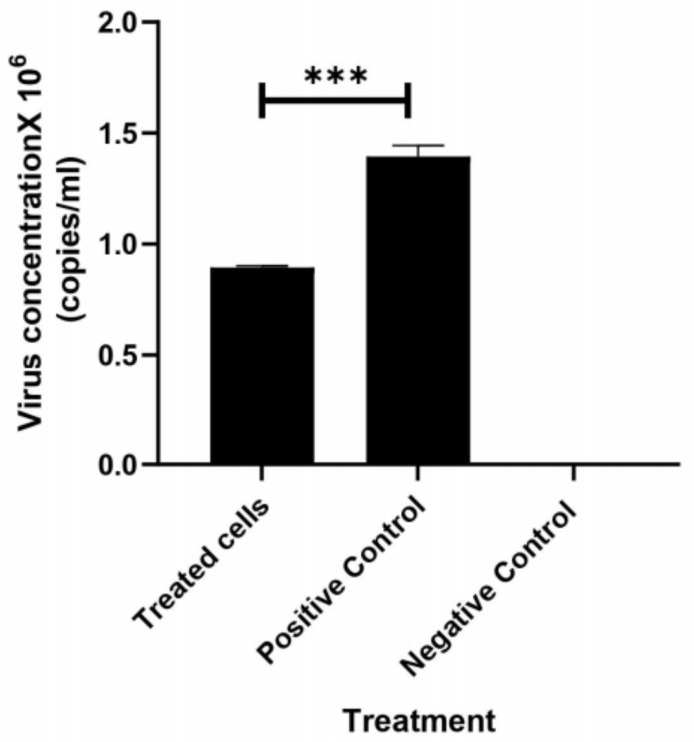
Quantification of HCV viral replication inhibition in PBMCs using RT-qPCR. All data are expressed as mean ± SEM and considered significantly different at *p* < 0.0005 ***.

The ability of silk protein to inhibit HCV replication on PBMCs was quantified using qPCR. The obtained results indicated that silk protein at 100 µg/mL showed the ability to inhibit HCV replication on the PBMC model by 66.2% via reducing the viral load from 1.36 × 10^6^ to 0.9 × 10^5^ copies/mL (Figure 5 and Table 1).

#### 3.2.3. Quantification of the Induced ROS Using Flow Cytometry in HCV-Infected Model

**Figure 6:**
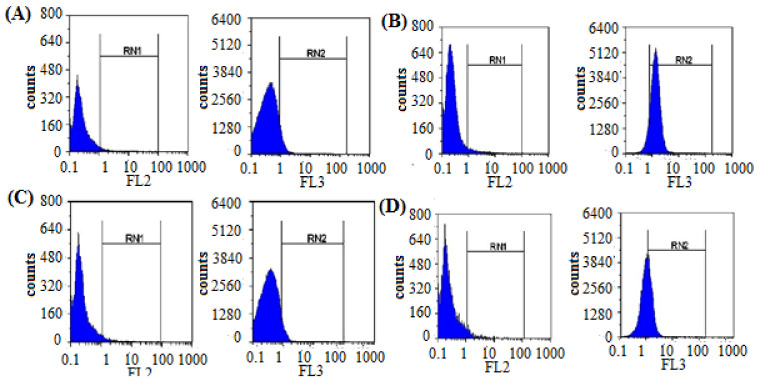
The flow cytometry analysis of the cellular induced ROS in untreated control cells (**A**), positive control HCV infected cells (**B**), silk-protein-treated cells (**C**), and silk-protein-treated HCV-infected cells (**D**). RN1 is the gating region for parameter number 1 using red laser and RN2 is the gating region for parameter number 1 using blue laser.

**Figure 7:**
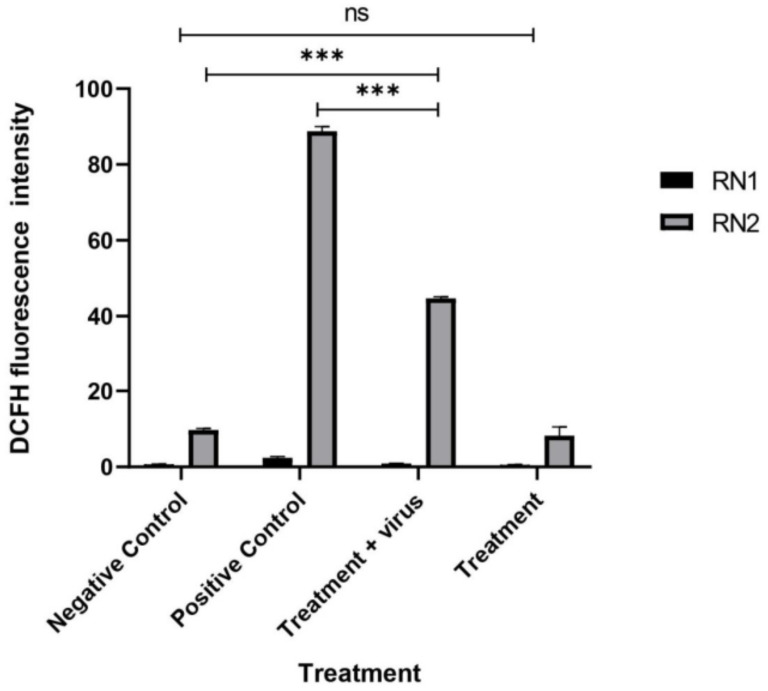
The fluorescence intensity in HCV-infected PBMC models after silk protein treatment. All data are expressed as mean ± SEM and considered significantly different at *p* < 0.05 *, *p* < 0.005 **, *p* < 0.0005 ***.

The induced cellular ROS in PBMCs after HCV infection and treatment were quantified using flow cytometry (Figure 6). The obtained results indicated a great induction of cellular ROS in PBMCs after HCV infection (87.9) compared with the uninfected cells (9.5). After treatment, silk protein dramatically reduced the induced ROS from 87.9 to 44.5 with 50.6% inhibition (Figure 7). It is also worth mentioning that silk protein did not induce a significant ROS induction (6.6) in PBMCs compared with the negative untreated cells. 

#### 3.2.4. 3D Predicted Structure Models

**Figure 8:**
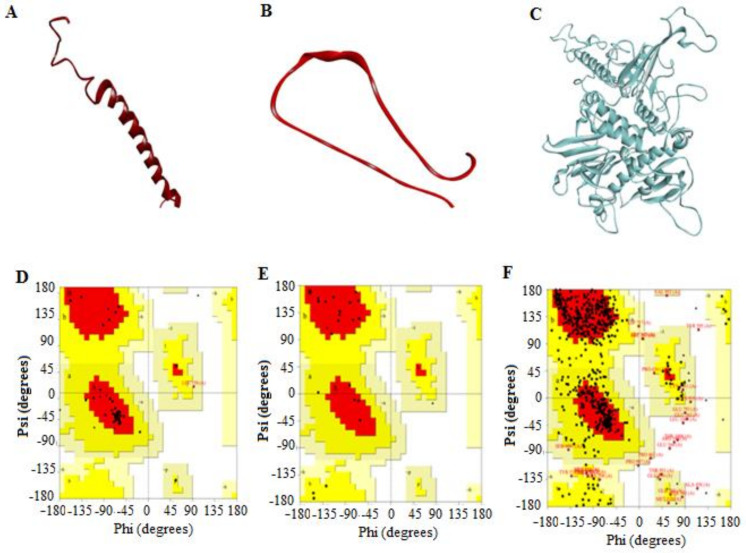
The 3D predicted structures of the silk protein subunits, fibroin and sericin, and ADV7 polymerase and their validation. (**A**–**C**) Backbone structure of silk fibroin, sericin, and ADV7 polymerase, respectively, as given by Swiss-Model protein-modeling server (https://swissmodel.expasy.org/ (accessed on 13 October 2021). (**D**–**F**) Ramachandran plots of silk protein fibroin, sericin, and ADV7 polymerase, respectively.

The 3D structures of the two silk protein subunits and ADV7 polymerase were modeled by the Swiss-Model server. The protein sequences in Fast Adaptive Shrinkage Threshold Algorithm (FASTA) format were submitted to this online tool to supply the most accurate predictions for their structure. Model 1, with the highest quality and a good C-score value, was established (Figure 8A–C). The molecular weight values of the *Bombyx mori* silk fibroin and sericin 1A’ were computed using the Expasy online server. The results showed a great similarity between them (27.6 and 69.9 kDa, respectively) and those obtained from *Bacillus* sp. (30 and 66 kDa, respectively) in our recently published work [10]. Therefore, these two types of *Bombyx mori* silk protein subunits were chosen here for the computational studies.

The quality of the predicted structures was confirmed by the Ramachandran plot (Figure 8D–F), which analyzed psi (ψ) and phi (F) torsion angles of the structural backbone. The residues of the ADV7 polymerase 3D structure in the most favored, allowed, and disallowed regions were 74.7%, 24.0%, and 1.3%, respectively. For the 3D structure of silk fibroin, these values were 89.5%, 10.5%, and 0%, respectively. For the 3D structure of silk sericin, these values were 76.2%, 23.8%, and 0%, respectively. The overall G-factors (measurement of unusualness for main-chain dihedral angles and covalent forces) of the predicted models of ADV7 polymerase, fibroin, and sericin were −0.41, −0.27, and −0.31, respectively. These results indicated that the dihedral angles, ψ and F, in the selected model backbone were reasonably accurate.

### 3.3. The Predicted Inhibitory Mechanism of Silk Fibroin and Sericin on ADV7, HCV, and HSV-1 Polymerases

**Figure 9:**
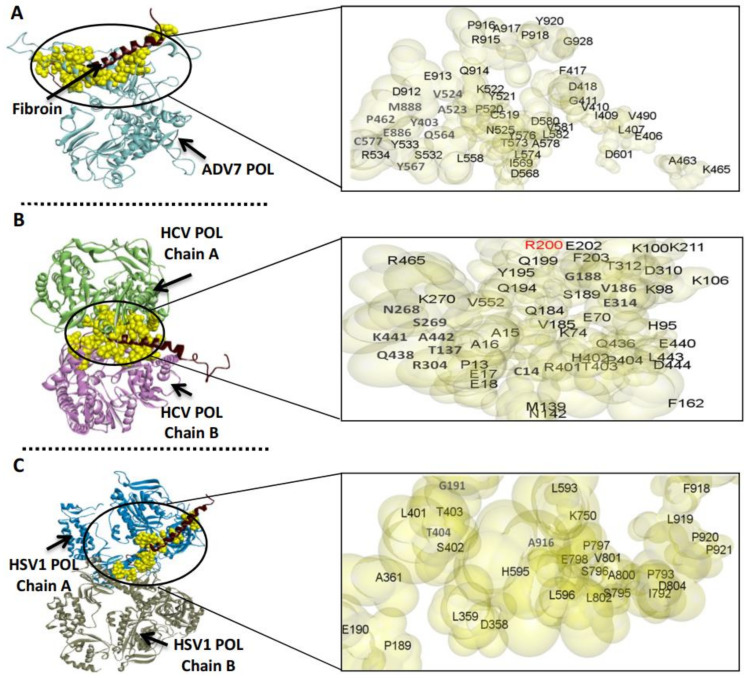
Molecular docking of ADV7, HCV, and HSV-1 polymerase (POL) and silk fibroin. (**A**–**C**) The docked complexes of ADV7 POL (shown in light blue), HCV POL (shown in light green “chain A” and purple “chain B”), and HSV-1 POL (shown in blue “chain A” and gray “chain B”) with fibroin (shown in dark red), respectively, as provided by the GRAMM-X Protein-Protein Docking platform and visualized by Discovery Studio software. The interacting pocket residues of the docked complex are indicated by yellow space-filling spheres style, magnification of these regions shows the interface residues on the viral polymerase (shown in pale yellow-gray surface), and the red-colored residue represents the matched residue with the enzyme active site.

**Figure 10:**
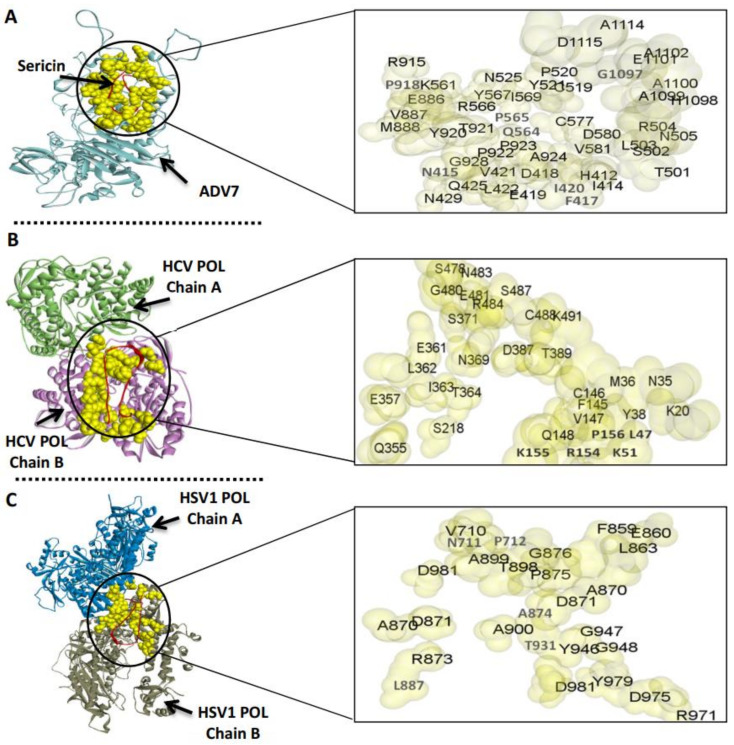
Molecular docking of ADV7, HCV, and HSV-1 polymerase (POL) and silk sericin. (**A**–**C**) The docked complexes of ADV7 POL (shown in light blue), HCV POL (shown in light green “chain A” and purple “chain B”), and HSV1 POL (shown in blue “chain A” and gray “chain B”) with sericin (shown in dark red), respectively, as provided by the GRAMM-X Protein-Protein Docking platform and visualized by Discovery Studio software. The interacting pocket residues of the docked complex are indicated by yellow space-filling spheres style, and magnification of these regions shows the interface residues on the viral polymerase (shown in pale yellow-gray surface).

To predict the inhibitory mechanism of the silk protein on the polymerase activity of the target viruses, the silk fibroin or sericin was docked separately with the viral polymerase (Figure 9 and Figure 10). Then, the docked complex was analyzed by the PDBePISA tool to explore the binding affinity, interface residues, and other details. The outcomes of PDBePISA showed that fibroin interacted with chain A of ADV7 polymerase (47 res, 6 hydrogen bonds) and both chains A and B of HCV (48 res, 4 hydrogen bonds) and HSV-1 (29 res, 3 hydrogen bonds) polymerases. Sericin bound to chain A of ADV7 polymerase (48 res, 7 hydrogen bonds), chain B of HCV polymerase (32 res, 11 hydrogen bonds), and chains A and B of HSV-1 polymerase (27 res, 4 hydrogen bonds). Furthermore, the binding affinity of fibroin and sericin to the studied polymerases was deduced from the predicted ΔG values. These values were −23.0 (ADV7 polymerase), −11.2 (HCV polymerase), and −14.4 kcal/mol (HSV-1 polymerase) for fibroin and −17.6, −10.5, and −9.0, respectively, for sericin. The ΔG *p*-value was also provided by the PDBePISA and indicated that the interface surface in the studied fibroin– or sericin–polymerase docked complexes was interaction-specific (*p*-value < 0.5). The active site residues of ADV7, HCV, and HSV-1 polymerases were compared with the interface residues in the fibroin– or sericin–polymerase docked complexes. The results showed that either fibroin or sericin interacted with the active site residues of the examined viral polymerases, except for HCV polymerase, for which fibroin bound to R200A only (Figure 9B).

## 4. Discussion

There are essential requirements for the discovery and exploration of novel natural agents against both RNA and DNA viruses. A variety of natural compounds derived from microorganisms and plants as medicinal products have been investigated for the management and control of numerous viral diseases. Silk is considered one of the most important natural fibrous proteins and is mainly obtained from animal origins, including silkworms and spiders. Therefore, the most popular studies in this field have focused on the production of polymeric silk proteins from animal origins or using genetically engineered bacteria [53,54,55]. We identified a previously isolated bacterial strain from petroleum oil origin called *Bacillus sp. strain NE (MK231249)* with the ability to form an exo-biopolymeric extract. Using molecular identification and exo-polymer chemical and physical characterizations, this bacterial exo-polymeric extract was identified as a bacterial silk-like protein [29]. In a previous study, El-Fakharany et al. revealed that the extracted sericin from bacterial silk was found to be like that isolated from animal origin, with potent biological activities, including antioxidant, anticancer, and antibacterial activities [10]. Silk proteins of animal origin have many biological and pharmacological properties, including antioxidant, antitumor, antimicrobial, and anti-inflammatory functions. The available studies on silk proteins show that these proteins may reduce the free radicals in the surrounding media and contamination by microbial pathogens such as bacteria and fungi. Therefore, silk proteins can be used in numerous applications, such as in the treatment of fabrics for medicinal uses and the preparation of wound healing gels. A particular coating of sericin on polyamide or polyester had been used as media for air filters, which were found to assist in sanitizing the contaminated air [9,52,53]. In the literature, there are several studies about the investigation of the antibacterial and antifungal activities of silk proteins, but reports on their antiviral properties are relatively rare. The present study, for the first time, was aimed to investigate the action of bacterial silk-like proteins against different types of viruses. For this purpose, the whole bacterial silk-like proteins were checked for their potential antiviral activity against HSV-1, ADV7, and HCV infectivity in Vero cells and PBMCs. The obtained results revealed that the bacterial silk-like proteins can display a direct virucidal effect on HSV-1 and ADV7, which might be through their amino acid structure and the action of secondary metabolites such as polyphenols and flavonoids [8]. In fact, the bacterial silk-like proteins were able to inhibit and neutralize the infection of HSV-1 and ADV7 upon entry into Vero cells with IC_50_ values of 4.1 ± 0.7 μg/mL and 46.4 ± 0.5 μg/mL, respectively. At similar conditions, the IC_50_ values of adsorption inhibitory effect on HSV-1 and ADV7 were estimated to be 12.5 ± 0.1 μg/mL and 222.4 ± 1.0 μg/mL, respectively. Furthermore, the bacterial silk-like proteins showed potent antireplication activities against HSV-1 and ADV7 with IC_50_ values of 9.8 ± 0.3 μg/mL and 109.3 ± 2.1 μg/mL, respectively. Results of the present study indicated that the bacterial silk proteins had the ability to eliminate ADV7 and HSV-1 by direct virucidal activity determined to be 85.8% and 89.9%, respectively, using qPCR and MTT methods. We showed that the antireplication effect of these proteins exhibited viral elimination of about 63.2% and 77.5%, respectively. However, ribavirin and acyclovir exhibited an antireplicative effect determined to be 77.9% and 62.9%, respectively. On the other hand, the bacterial silk proteins showed inhibitory mechanisms of viral polymerases with IC_50_ estimated to be 164.1 ± 11.1 μg/mL and 11.8 ± 0.6 μg/mL against ADV7 and HSV-1, respectively, similar to the inhibitory effect of the standard drugs.

For the anti-HCV inhibitory effect of bacterial silk proteins, the replication of HCV was inhibited by 66.2% inside infected PBMCs, as determined using RT-qPCR technique, at a concentration of 100 µg/mL. Furthermore, the bacterial silk proteins showed a potent reduction in cellular ROS inside the HCV-infected PBMCs with an inhibition percentage of 50.6%, as compared to untreated HCV-infected cells which showed ROS activity determined to be 87.9%. The docking analysis revealed that silk fibroin and sericin can interact with ADV7, HSV-1, and HCV polymerases by hydrogen bonds and salt bridge interactions, and the interface surface in the obtained docked complexes is interaction-specific (Δ^i^G *p*-values < 0.5). From the Δ^i^G values, we can deduce that the binding affinity of silk fibroin to ADV7 and HSV-1 polymerases was greater than that of silk sericin. Both silk proteins had nearly the same binding affinity to the HCV polymerase. The computational findings also demonstrated the inability of either silk fibroin or sericin to bind to the predicted active site residues of ADV7, HSV-1, or HCV polymerases. So, these viral polymerases can be inhibited by silk fibroin or sericin in an uncompetitive or noncompetitive manner [56]. This can be attributed to the distinctive capability of binding regions to be engaged in multiple interactions with various binding partners of bacterial silk proteins. One of the main possible mechanisms for this antiviral potential could be related to the relatively high cationic nature of the silk polymer, which enables it to bind to the infected host cells and block the viral particles from entry. 

The biological activities of silk-like proteins are attributed to their unique composition of amino acids; in particular, their structures contain hydroxyl groups of serine and threonine, which chelate many essential elements like iron. In addition, silk sericin has many aromatic amino acids in its structure, which provide an electron-donating property, besides the action of secondary metabolites such as flavonoids and polyphenols [8,57]. Furthermore, alteration in pH of protein during the extraction process (e.g., using base or acid environment with heating) changes the ionization of amino acid, consequently producing sericin proteins with different lengths which contain large proportions of β-sheets, α-helix, turns, and random coils [58]. Additionally, percentages of random coils and β-sheets reflect the amorphous nature or crystallinity of sericin protein, respectively [59]. Moreover, the bacterial silk-like protein was found to contain unique amino acids such as glycine and proline and contain aliphatic hydrophobic amino acids such as alanine, valine, leucine, and isoleucine with overall composition of 32% [29]. The bacterial silk-like biopolymer can be developed into medicinally useful lead candidates for antiviral therapeutics and control.

## 5. Conclusions

The results obtained from the present study confirm that the bacterial silk-like proteins have potent antiviral activity against both DNA and RNA viruses. Considering the other biomedical properties and various biotechnological uses of bacterial silk-like proteins, our findings establish the further significance of these proteins as a biopharmaceutic candidate that may be incorporated with other potent drugs for delivery enhancement and achievable treatment. In vitro studies revealed that the bacterial silk-like proteins showed efficient antiviral activities using many molecular mechanisms with high safety on normal cells. Consequently, these results indicate that bacterial silk-like proteins can also be widely applied in controlling and managing viral infection and pandemics (especially in the control of COVID-19) alone or incorporated with other viral drugs. Furthermore, the demand for efficient antiviral drugs with high safety might prompt the consideration of the use of the bacterial silk proteins as a potent candidate in medicinal applications.

## Figures and Tables

**Table 1:** Determination of HCV viral count in PBCs using RT-qPCR.

Samples	Fluor	Cq	Virus Conc. (Copies/mL)	Inhibition%
Treated cells	SYBR	23.89	0.9 × 10^5^	66.2
Positive control	SYBR	28.33	1.36 × 10^6^	0.0
Negative control	SYBR	4.69	-	-

## Data Availability

The data presented in this study are available on request from the corresponding author.
